# Atypical Presentation of Multidrug-Resistant Acinetobacter baumannii Pneumonia in a Post-Surgical ICU Patient: A Case Report

**DOI:** 10.7759/cureus.89059

**Published:** 2025-07-30

**Authors:** Zaina Rawashdeh, Marah J Alshatarat, Basil G Daradkeh, Dina Awwad, Sara H Sawalha

**Affiliations:** 1 Medicine, University of Jordan, Amman, JOR; 2 General Practice, Jordan University of Science and Technology, Amman, JOR; 3 Medicine, Mutah University, Irbid, JOR

**Keywords:** acinetobacter baumannii, colistin, icu (intensive care unit), multidrug resistance, pneumonia, post-surgical infection, tigecycline

## Abstract

*Acinetobacter baumannii* is a Gram-negative opportunistic pathogen increasingly encountered in hospital environments, particularly in intensive care units (ICUs), where it presents a major clinical challenge due to its multidrug-resistant (MDR) profile. Its resistance to multiple antibiotic classes makes it a serious therapeutic challenge, particularly in post-surgical patients where symptoms may be atypical. We report a case of a previously healthy 64-year-old male patient with a history of hypertension and coronary artery disease, admitted to the ICU following elective abdominal aortic aneurysm repair. On postoperative day five, he developed mild respiratory symptoms, low-grade fever, and oxygen desaturation, but with no abnormal lung auscultation findings. Elevated inflammatory markers and radiologic evidence of faint bilateral perihilar infiltrates and subtle ground-glass opacities prompted further investigation. Cultures revealed MDR *A. baumannii* resistant to carbapenems, aminoglycosides, and fluoroquinolones, but susceptible to colistin and tigecycline. The patient received combination intravenous therapy with colistin and tigecycline, resulting in rapid clinical improvement, normalization of inflammatory markers, and complete radiologic resolution by day 14. This case underscores the importance of maintaining a high index of suspicion for MDR pneumonia in post-surgical ICU patients with non-specific symptoms and highlights the role of early diagnosis and targeted therapy in achieving favorable outcomes.

## Introduction

*Acinetobacter baumannii* is a Gram-negative, non-fermenting coccobacillus frequently associated with hospital-acquired infections, especially in critically ill patients in intensive care units (ICUs) [1]. Its ability to persist in the hospital environment and acquire resistance to multiple antibiotic classes, including carbapenems, aminoglycosides, and fluoroquinolones, has led to the emergence of multidrug-resistant (MDR) and extensively drug-resistant (XDR) strains [2,3]. These resistant strains significantly limit treatment options and are associated with increased mortality, particularly in post-surgical ICU settings due to their virulence and environmental hardiness [4].

Pneumonia caused by *A. baumannii* remains a leading cause of ICU-acquired infections worldwide [5]. Global mortality from carbapenem-resistant *A. baumannii* (CRAB) infections remains high, with 28-day mortality rates up to 53%, particularly in ICU patients [5]. Local prevalence in Jordan has risen to over 90% resistance in ICU isolates, highlighting the urgent need for early detection and effective treatment strategies [6]. While the classic presentation includes fever, leukocytosis, purulent sputum, and lobar consolidation on imaging, atypical cases, particularly in sedated or immunocompromised patients, may lack clear clinical or radiographic indicators. Given the organism’s rapid disease progression and resistance profile, early recognition and tailored treatment are essential.

Patients with CRAB bacteremia had significantly higher in-hospital mortality compared to those with other Gram-negative bacteremias: 35% vs. 21% at 14 days, 53% vs. 30% at 28 days, and 74% vs. 52% overall [7]. Multivariable competing risk regression analysis confirmed that CRAB bacteremia was independently associated with an increased risk of 28-day inpatient death (HR: 1.80, 95% CI: 1.28-2.54) [8]. A hospital-based study in Jordan reported a rise in resistance rate from 14% in 2010 to over 35% by 2020, with recent reports in 2023-2024 indicating rates exceeding 90% in ICU isolates [9]. We present a case illustrating an unusual and subtle presentation of MDR *A. baumannii* pneumonia in a post-surgical ICU patient, highlighting the need for clinical vigilance.

## Case presentation

A 64-year-old male patient with a medical history of controlled hypertension and stable coronary artery disease was admitted to the ICU following elective open repair of an abdominal aortic aneurysm. He was a non-smoker and had no history of chronic pulmonary disease, recent infection, immunosuppressive therapy, or hospital admission within the past six months. There was also no recent travel history. The patient remained afebrile and asymptomatic during the first four postoperative days, with no cough, dyspnea, or chest discomfort. Initial vital signs and laboratory investigations, including inflammatory markers and white blood cell counts, were within normal limits (Table 1). No signs of systemic infection were evident.

**Table 1:** Laboratory Parameter Changes in Hemodynamic, Respiratory, and Inflammatory Markers From Baseline to Diagnosis of Multidrug-Resistant Acinetobacter baumannii Pneumonia in an ICU Patient ICU: intensive care unit; WBC: white blood cell

Parameter	ICU admission	Day 5 (pneumonia onset)	Reference range
Vital signs			
Temperature (°C)	36.8	38	36.5–37.5
Heart rate (beats/min)	78	102	60–100
Blood pressure (mmHg)	130/80	125/78	90/60–120/80
Respiratory rate (breaths/min)	16	24	12–20
SpO₂ (%)	98 (room air)	92 (2 L/min oxygen)	>95 (room air)
PaO₂ (mmHg)	90	65	80–100
Complete blood count			
WBC (×10³/μL)	8.9	12.3	4.0–11.0
Neutrophils (%)	60	85	40–70
Hemoglobin (g/dL)	13.2	12.5	13.5–17.5
Hematocrit (%)	39	37	41–53
Platelets (×10³/μL)	220	210	150–450
Inflammatory markers			
C-reactive protein (mg/L)	12	75	<5
Procalcitonin (ng/mL)	0.1	2.5	<0.5

On ICU day five, the patient developed low-grade fever, tachypnea, tachycardia, mild hypoxemia, and increased work of breathing. Lung auscultation remained normal; a minimally productive cough was observed, producing scant whitish non-purulent sputum. Arterial blood gas analysis showed a decline in oxygenation. These clinical changes were accompanied by abnormal laboratory findings and significantly elevated inflammatory markers (Table 1).

Despite the absence of abnormal auscultation findings, the overall clinical picture raised suspicion of hospital-acquired pneumonia. The patient was initiated on empirical broad-spectrum antibiotic therapy consisting of intravenous piperacillin-tazobactam and levofloxacin. Further diagnostic workup, including chest X-ray, high-resolution computed tomography (HRCT), and sputum culture, was performed to confirm the diagnosis and guide appropriate management.

**Figure 1:**
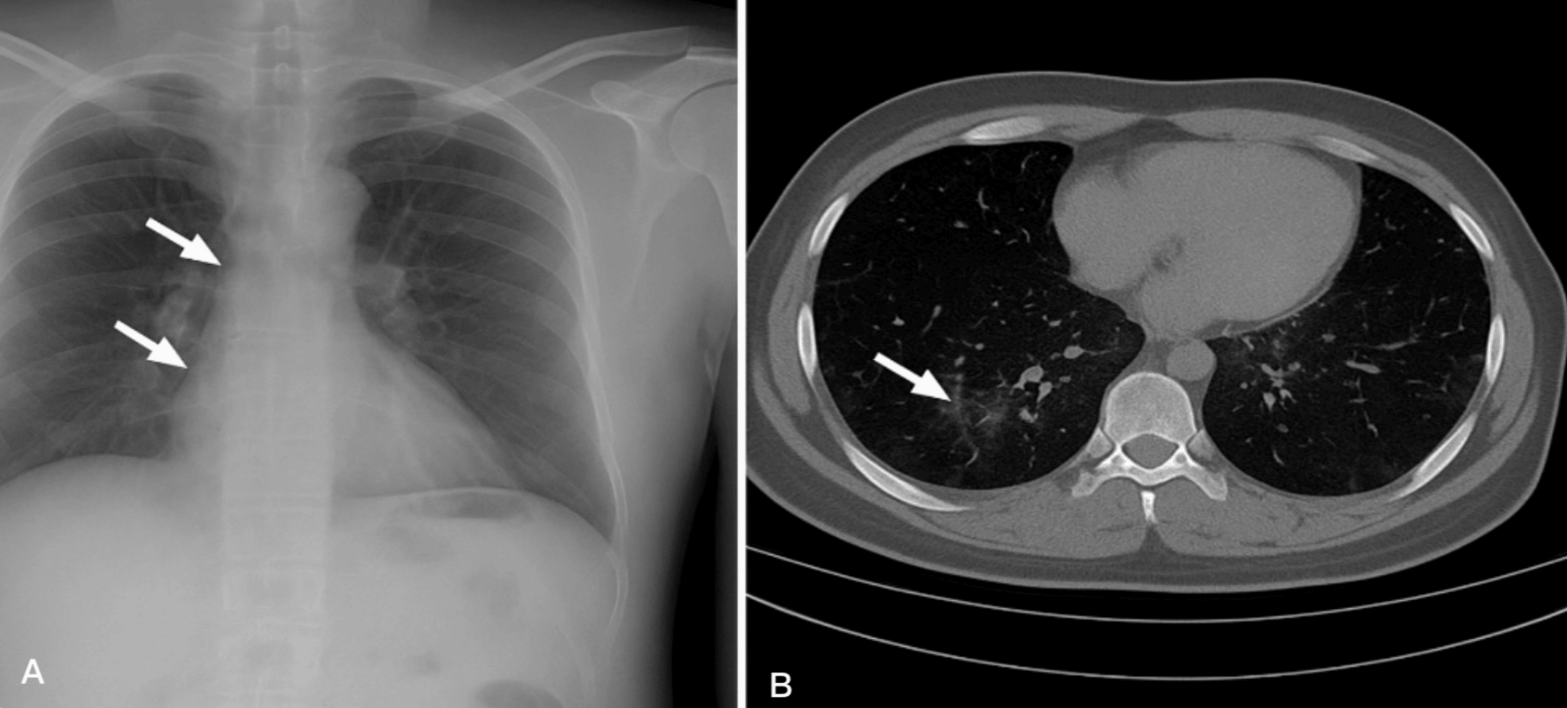
Radiographic and High-Resolution Computed Tomography (HRCT) Findings in a Postoperative ICU Patient With MDR Acinetobacter baumannii Pneumonia (A) Frontal chest radiograph shows ill-defined opacities in the right lower and mid-lung zones (white arrows), suggestive of localized consolidation. (B) Axial CT scan of the chest confirms patchy consolidation with surrounding ground-glass opacities in the right lower lobe (white arrow), without pleural effusion. These findings are consistent with a focal atypical pneumonia, later confirmed microbiologically as multidrug-resistant (MDR) *Acinetobacter baumannii* infection in a post-surgical intensive care unit (ICU) patient

Chest X-ray demonstrated faint bilateral perihilar infiltrates without evidence of consolidation. HRCT revealed patchy ground-glass opacities and mild interstitial thickening, without abscess or effusion (Figure 1). A spontaneous sputum sample was collected by deep cough using a sterile technique to reduce contamination risk. No induction was required. Blood cultures were not obtained as the patient remained hemodynamically stable, with no signs of bacteremia or sepsis per institutional guidelines. Within 48 hours, sputum cultures identified MDR A. *baumannii*, resistant to carbapenems, aminoglycosides, fluoroquinolones, and beta-lactams, but susceptible to colistin and tigecycline (Table 2). Based on the susceptibility profile, therapy was de-escalated to intravenous colistin (loading dose 9 million IU, followed by 4.5 million IU every 12 hours) and tigecycline (loading dose 100 mg, then 50 mg every 12 hours); renal function and serum creatinine were monitored every 48 hours; no nephrotoxicity was observed during colistin therapy. Therapy was de-escalated from empiric broad-spectrum to culture-guided combination therapy. Upon identification of MDR *A. baumannii*, strict contact isolation was implemented per ICU infection control policy, including use of gowns, gloves, and dedicated equipment to prevent cross-transmission.

**Table 2:** Antibiotic Susceptibility Profile of Acinetobacter baumannii Isolate

Antibiotic	Susceptibility	Resistance rate (%)
Carbapenems (imipenem, meropenem)	Resistant	90%–95%
Piperacillin–tazobactam	Resistant	85%–90%
Aminoglycosides (amikacin, gentamicin)	Resistant	70%–80%
Fluoroquinolones (ciprofloxacin, levofloxacin)	Resistant	75%–85%
Colistin	Susceptible	5%–10%
Tigecycline	Susceptible	15%–20%
Sulbactam	Intermediate	Variable (30%–50%)

The patient showed a favorable response to targeted therapy. Fever resolved within 72 hours, inflammatory markers steadily declined, and oxygenation progressively improved. Follow-up chest imaging revealed partial resolution of infiltrates. By day 14 of therapy, laboratory markers of infection had normalized (Table 3). The patient was transferred to the surgical ward and later discharged in stable condition, with no respiratory sequelae or relapse during the hospitalization.

**Table 3:** Patient’s Inflammatory and Oxygenation Parameters at Pneumonia Onset and Throughout Colistin–Tigecycline Therapy WBC: white blood cell

Parameter	Day 0	Day 3	Day 7	Day 14	Reference range
Temperature (°C)	38.0	37.2	36.8	36.5	36.5–37.5
WBC (×10³/μL)	12.3	9.8	7.6	6.8	4.0–11.0
C-reactive protein (mg/L)	75	32	10	3	<5
Procalcitonin (ng/mL)	2.5	1.1	0.4	0.1	<0.5

## Discussion

*A. baumannii* is a highly resilient nosocomial pathogen that thrives in ICU environments, where prolonged hospital stays, mechanical ventilation, and invasive procedures create an ideal setting for infection [10]. Its capacity to develop multidrug resistance, particularly to carbapenems, aminoglycosides, and fluoroquinolones, has made it a major contributor to morbidity and mortality in critical care settings [11]. Its ability to survive on dry surfaces and resist disinfectants further complicates infection control efforts [12]. This case was atypical as the patient exhibited only mild respiratory symptoms, normal auscultation findings, and faint imaging changes, which contrast with typical MDR pneumonia presentations characterized by overt respiratory distress, lobar consolidation, and purulent sputum.

*A. baumannii* pneumonia typically presents with overt respiratory symptoms and characteristic radiological findings; our case illustrates a subtle and delayed presentation following elective surgery. This aligns with growing evidence that post-surgical or sedated patients may mount blunted clinical responses due to altered immune function, impaired mucociliary clearance, and the suppressive effects of sedatives and analgesics [13]. Similar cases in ICU literature highlight how mild respiratory signs and ambiguous imaging findings can delay diagnosis [13]. Timely use of inflammatory markers such as C-reactive protein (CRP) and procalcitonin was critical in recognizing infection despite the lack of classic features [14]. Elevated procalcitonin levels, in particular, have been associated with bacterial infection severity and may aid early decision-making in critically ill patients [14].

The pathogen’s resistance profile in this case was consistent with global surveillance data, which continue to report rising rates of CRAB, leaving few effective antibiotics. Sulbactam has shown efficacy in the treatment of *A. baumannii* infections, but its activity against carbapenem-resistant isolates is diminishing [15]. Sulbactam-durlobactam shows great potential in treating MDR *A. baumannii* infections. It continued to be effective, even for isolates unresponsive to sulbactam, carbapenems, and cefiderocol [15]. Sulbactam-based combination therapy continues to be a key component of targeted treatment strategies, as sulbactam acts as a β-lactamase inhibitor with intrinsic activity against *Acinetobacter* species [16]. Newer agents, such as cefiderocol and sulbactam-durlobactam, show promise in refractory CRAB cases but were unavailable locally, underscoring the limited therapeutic arsenal in many resource-limited ICU settings. CRAB has been increasingly encountered since the early 2010s, with a notable surge in ICU-related outbreaks reported from 2012 onward, particularly in regions with high antibiotic pressure [17]. Recent studies confirm that CRAB remains prevalent across multiple regions, with increasing detection in both endemic and epidemic ICU settings [18].

Our patient’s isolate remained susceptible to colistin and tigecycline, enabling the use of a combination therapy that has demonstrated clinical success in multiple reports [19]. Mortality benefits of such regimens remain debated; microbiological clearance rates are generally improved [19]. Early microbiological sampling and therapy de-escalation based on culture results helped minimize unnecessary broad-spectrum antibiotic use and reduced the risk of nephrotoxicity associated with colistin [20]. These targeted strategies are integral to antimicrobial stewardship programs and have been associated with improved clinical outcomes and reduced resistance pressures [20].

While the patient’s clinical response was favorable, the absence of long-term follow-up limits conclusions regarding recurrence and long-term treatment outcomes and the initiation of truly individualized treatment strategies, such as de-escalation based on susceptibility results, resulting in improved therapeutic efficacy. Further studies are needed to explore relapse risk and long-term sequelae in CRAB pneumonia cases.

## Conclusions

MDR *A. baumannii* pneumonia in critically ill post-surgical patients may present atypically, with minimal clinical signs and subtle imaging features. The global rise in antimicrobial resistance, driven by widespread antibiotic misuse, has increased the mortality risk associated with these infections. Early detection, prompt microbiological diagnosis, and individualized antimicrobial therapy are essential to improving clinical outcomes. This case highlights the effectiveness of colistin-tigecycline therapy following the failure of broad-spectrum antibiotics. Given the risk of underdiagnosis, clinicians must maintain a high index of suspicion for atypical presentations. Multidisciplinary coordination and strict adherence to antimicrobial stewardship programs are critical for managing MDR pathogens in intensive care settings, as their early and accurate recognition enables the initiation of truly individualized treatment strategies, resulting in improved therapeutic efficacy, minimized adverse effects, significantly enhanced quality of life, better prognosis, and long-term outcomes.
